# Effect of Estrogen on Heteronemin-Induced Anti-proliferative Effect in Breast Cancer Cells With Different Estrogen Receptor Status

**DOI:** 10.3389/fcell.2021.688607

**Published:** 2021-07-26

**Authors:** Yu-Chen S. H. Yang, Zi-Lin Li, Tung-Yung Huang, Kuan-Wei Su, Chi-Yu Lin, Chi-Hung Huang, Han-Yu Chen, Mei-Chin Lu, Haw-Ming Huang, Sheng-Yang Lee, Jaqueline Whang-Peng, Hung-Yun Lin, Paul J. Davis, Kuan Wang

**Affiliations:** ^1^Joint Biobank, Office of Human Research, Taipei Medical University, Taipei, Taiwan; ^2^Graduate Institute of Nanomedicine and Medical Engineering, College of Medical Engineering, Taipei Medical University, Taipei, Taiwan; ^3^Graduate Institute of Cancer Biology and Drug Discovery, College of Medical Science and Technology, Taipei Medical University, Taipei, Taiwan; ^4^Department of Dentistry, Hsinchu MacKay Memorial Hospital, Hsinchu, Taiwan; ^5^School of Dentistry, College of Oral Medicine, Taipei Medical University, Taipei, Taiwan; ^6^Division of Cardiology, Department of Internal Medicine, Cathay General Hospital, Taipei, Taiwan; ^7^National Museum of Marine Biology and Aquarium, Pingtung, Taiwan; ^8^Graduate Institute of Marine Biology, National Dong Hwa University, Pingtung, Taiwan; ^9^Center for Tooth Bank and Dental Stem Cell Technology, Taipei Medical University, Taipei, Taiwan; ^10^Department of Dentistry, Wan-Fang Medical Center, Taipei Medical University, Taipei, Taiwan; ^11^Cancer Center, Wan Fang Hospital, Taipei Medical University, Taipei, Taiwan; ^12^TMU Research Center of Cancer Translational Medicine, Taipei Medical University, Taipei, Taiwan; ^13^Traditional Herbal Medicine Research Center of Taipei Medical University Hospital, Taipei Medical University, Taipei, Taiwan; ^14^Pharmaceutical Research Institute, Albany College of Pharmacy and Health Sciences, Rensselaer, NY, United States; ^15^Department of Medicine, Albany Medical College, Albany, NY, United States

**Keywords:** heteronemin, estrogen, breast cancer, estrogen receptor status, anti-proliferation, mitochondrial ROS

## Abstract

Estrogen (E_2_) has multiple functions in breast cancers including stimulating cancer growth and interfering with chemotherapeutic efficacy. Heteronemin, a marine sesterterpenoid-type natural product, has cytotoxicity on cancer cells. Breast cancer cell lines, MCF-7 and MDA-MB-231, were used for investigating mechanisms involved in inhibitory effect of E_2_ on heteronemin-induced anti-proliferation in breast cancer cells with different estrogen receptor (ER) status. Cytotoxicity was detected by cell proliferation assay and flow cytometry, gene expressions were determined by qPCR, mechanisms were investigated by Western blot and Mitochondrial ROS assay. Heteronemin exhibited potent cytotoxic effects against both ER-positive and ER-negative breast cancer cells. E_2_ stimulated cell growth in ER-positive breast cancer cells. Heteronemin induced anti-proliferation via suppressing activation of ERK1/2 and STAT3. Heteronemin suppressed E_2_-induced proliferation in both breast cancer cells although some gene expressions and anti-proliferative effects were inhibited in the presence of E_2_ in MCF-7 and MDA-MB-231 cells with a higher concentration of heteronemin. Heteromenin decreased the *Bcl-2/Bax* ratio to inhibit proliferation in MDA-MB-231 but not in MCF-7 cells. Both heteronemin and E_2_ increased mitochondrial reactive oxygen species but combined treatment reversed superoxide dismutase (SOD)s accumulation in MCF-7 cells. Heteronemin caused G_0_/G_1_ phase arrest and reduced the percentage of cells in the S phase to suppress cancer cell growth. In conclusion, Heteronemin suppressed both ER-positive and ER-negative breast cancer cell proliferation. Interactions between E_2_ and heteronemin in signal transduction, gene expressions, and biological activities provide insights into the complex pathways by which anti-proliferation is induced by heteronemin in E_2_-replete environments.

## Introduction

Although early detection methods and effective treatments have been developed for breast cancer, it is still the most common diagnosed cancer among women (Bray et al., 2018). Despite advancements in diagnostics and systemic treatments, up to one-third of patients with breast cancer undergo a mastectomy as initial surgical treatment to achieve local control (Anampa et al., 2015). Adjuvant systemic treatment, including chemotherapy, reduces the risks of distant recurrence and breast cancer mortality.

Generally, 17β-estradiol (E_2_) binds with estrogen receptor-α (ER-α) as a transcription factor complex to regulate expression of target genes and proteins that are important for biological functions (Hofseth et al., 1999). However, estrogen can initiate breast cancer development, and promotes breast cancer cell growth. Recently, we showed that estrogen is able to bind αvβ3, a cell surface integrin, to activate signal transduction and cancer growth (Cody et al., 2007); however, the mechanisms are not fully revealed. Although the ER-regulated signaling transduction pathway plays a vital role in breast cancer growth, it does not involve in breast cancer initiation (Cavalieri and Rogan, 2014). On the other hand, strong evidence indicates that estrogen oxidative metabolism may initiate breast cancer development majorly (Cavalieri et al., 1997; Devanesan et al., 2001).

Reactive oxygen species (ROS) include hydroxyl radicals (OH⋅), hydrogen peroxide H_2_O_2_, and superoxide (O_2_−) (Poillet-Perez et al., 2015). Both aerobic glycolysis and mitochondrial oxidative phosphorylation are cellular sources of ROS. During oxidation, electrons leak from the electron transport chain, transfer to oxygen, and convert approximately 1∼5% of the oxygen into superoxide (Bhat et al., 2015). However, additional electrons that prematurely leak from the respiratory chain under stressful conditions exacerbate superoxide production, thus causing detrimental effects (Poillet-Perez et al., 2015). At low concentrations, they are essential signaling molecules. On the other hand, high ROS quantities can cause damage to DNA and other macromolecules to trigger senescence (Cobbaut and Van Lint, 2018). High concentrations of ROS also permeabilize mitochondria leading to the release of cytochrome c which induces apoptosis (Cobbaut and Van Lint, 2018). Breast cancer cells modify metabolic pathways to facilitate increased proliferation and cell survival resulting in glucose and glutamine (Gwangwa et al., 2019). Cancer cells increase ROS production during cancer cell proliferation. Estrogen-mediated high ROS accumulation plays a key role in driving carcinogenesis (Tian et al., 2016). Excessive ROS serve as important effectors to increase genomic instability and activate redox-associated signaling pathways. Physiologically available concentrations of estrogens or estrogen metabolites that directly act on the mitochondria of mammary epithelial cells produce ROS, which subsequently enhances the phosphorylation of kinases to activate redox-sensitive transcription factors (Okoh et al., 2013). Therefore, ROS are important mediators of estrogen-induced cancer. Two metabolites of estrogen, 2-OHE1E_2_, and 4-OHE1E_2_, are highly redox-active and generate ROS in breast epithelial cells (Fussell et al., 2011). Long-term exposure to estrogen induces ROS overproduction and increases mitochondrial (mt)DNA mutations and mitochondrial protein damage. A recent study indicated that 4-OHE1E_2_ induces ROS and causes malignant transformation of MCF-10A cells. However, biological or chemical ROS scavengers prevent 4-OHE1E_2_-induced carcinogenesis in MCF-10A cells (Okoh et al., 2013). Excess ROS generated by repeated exposure to 4-OHE1(E2) caused malignancy of human mammary epithelial cells in nude mice (Okoh et al., 2013).

Heteronemin, the most abundant secondary metabolite in the sponge *Hippospongia* sp., exhibited potent cytotoxic activities against several cancer cell lines. It induced apoptosis in different types of cancer cells (Kollmann et al., 2015; Lee Y. S. et al., 2018; Lin et al., 2018; Cheng et al., 2019; Huang et al., 2020). It inhibited activation of extracellular signal-regulated kinase 1/2 (ERK1/2) and signal transducer and activator of transcription 3 (STAT3) (Huang et al., 2020). The role of nuclear factor (NF)-κB in heteronemin-induced anti-proliferation is controversial (Schumacher et al., 2010; Chen et al., 2018). It upregulated talin expression and talin phosphorylation in leukemia Molt4 cells (Chen et al., 2018). Heteronemin was shown to modulate mitochondrial (mt)ROS and oxidative phosphorylation (OXPHOS) (Cheng et al., 2019). Some studies indicated that heteronemin induces apoptosis via inhibition of the transforming growth factor (TGF)-β signal transduction pathway in cholangiocarcinomas (Lin et al., 2018). In addition, it inhibits p53 expression but does not affect apoptosis (Lin et al., 2018). Altogether, results have shown that this compound has potential as an anti-inflammatory and anti-cancer agent (Schumacher et al., 2010). Mitochondria are major sites for apoptosis, and they are highly regulated by the Bcl-2 family of proteins comprising both anti-apoptotic (Bcl-2 and Bcl-xL) and proapoptotic (Bax and Bak) members (el-Deiry et al., 1994; Chipuk et al., 2004; Papi et al., 2008; Weng et al., 2009; Xu et al., 2009; Gwangwa et al., 2019). Therefore, targeting mitochondria is a novel strategy for cancer therapy. Heteronemin was shown to target mitochondrial-mediated apoptosis (Wu et al., 2015; Chen et al., 2018; Cheng et al., 2019); however, evidence indicates there are other death pathways involved. Heteronemin induced a novel type of programmed cell death, “Ferroptosids” (Chang et al., 2021).

In the present study, we investigated the inhibitory effect of E_2_ on heteronemin-induced cytotoxic effects via suppressing activation of ERK1/2 and STAT3 in breast cancer cells. Heteronemin and E_2_ showed different effects on expressions of proliferation, angiogenesis, and growth factor receptor genes in breast cancer cells. E_2_ showed inhibitory effects on heteronemin-induced signal transduction, gene expression, and anti-proliferation in breast cancer cells. However, heteronemin induced anti-proliferation via different patterns in ER-positive and negative breast cancer cells. In the presence of E_2_, heteronemin induced anti-proliferation via modulating ROS in MCF-7 cells. On the other hand, it decreased *Bcl-2/Bax* ratio to inhibit cancer growth in MDA-MB-231 cells.

## Materials and Methods

### Cell Lines

Human ER-positive MCF-7 breast cancer cells (ATCC^®^ HTB-22^TM^), ER-negative MDA-MB-231 cells (ATCC^®^ HTB-26^TM^) and normal epithelial cell line Vero (ATCC^®^ CCL-81^TM^) was established from kidney of normal adult African green monkey were obtained from American Type Culture Collection (ATCC, Manassas, VA, United States). These cell lines were tested and authenticated by BCRC (isoenzyme analysis, *Mycoplasma*, cytogenetics, tumorigenesis, and receptor expression testing). Cells were maintained in Dulbecco’s modified Eagle’s medium (DMEM, Life Technologies, Carlsbad, CA, United States), supplemented with 10% fetal bovine serum (FBS). Incubation conditions were 5% CO_2_ at 37°C. Before the study, cells were placed in a 0.25% hormone-depleted serum-supplemented medium for 2 days.

### Cell Viability Assay

MCF-7, MDA-MB-231, and Vero cells were plated at a density of 4 × 10^3^ cells/well in 96-well plates. Cell viability was determined by using the Alamar Blue^®^ Assay Kit (Thermo Fisher Scientific, Watertown, MA, United States) at 72 h after treatment. Medium containing different drugs was replaced daily. At the time of detection, medium was removed, and cells were incubated with Alamar Blue^®^ reagent for 2 h at 37°C according to the manufacturer’s instructions. Plates were then analyzed using a VersaMax Microplate reader (Molecular Devices, San Jose, CA, United States) at a wavelength of 570 nm, with 600 nm as a reference.

### Real-Time Quantitative Polymerase Chain Reaction (qPCR)

Total RNA was extracted with genomic DNA removed with an Illustra RNAspin Mini RNA Isolation Kit (GE Healthcare Life Sciences, Buckinghamshire, United Kingdom). DNase I-treated total RNA (1 μg) was reverse-transcribed using a RevertAid H Minus First Strand cDNA Synthesis Kit (Life Technologies) into complementary (c)DNA. cDNAs were used as the template for the real-time PCR and analysis. Real-time PCRs were conducted using a QuantiNova^TM^ SYBR^®^ Green PCR Kit (Qiagen, Hilden, Germany) on a CFX Connect^TM^ Real-Time PCR Detection System (Bio-Rad Laboratories, Hercules, CA, United States). The reaction procedure involved initial denaturation at 95°C for 5 min, followed by 40 cycles of denaturing at 95°C for 5 s and combined annealing/extension at 60°C for 10 s, as shown in detail in the manufacturer’s instructions. Primer sequences are listed in Table 1. The relative gene expression (normalized to 18s reference gene) was calculated according to the ΔΔCT method. The fidelity of the PCR was determined by a melting temperature analysis.

**TABLE 1:** Primer sequences for the qPCR.

Name	Forward	Reverse
*CCND1*	5′-CAAGGCCTGAACCTGAGGAG-3′	5′-GATCACTCTGGAGAGGAAGCG-3′
*c-Myc*	5′-TTCGGGTACTGGAAAACCAG-3′	5′-CAGCAGCTCGAATTTCTTCC-3′
*PD-L1*	5′-GTTGAAGGACCAGCTCTCCC-3′	5′-ACCCCTGCATCCTGCAATTT-3′
*Bcl-2*	5′-TTGCCAGCCGGAACCTATG-3′	5′-CGAAGGCGACCAGCAATGATA-3′
*p21*	5′-CTGGGGATGTCCGTCAGAAC-3′	5′-CATTAGCGCATCACAGTCGC-3′
*Ki-67*	5′-GAAAGAGTGGCAACCTGCCTTC-3′	5′-GCACCAAGTTTTACTACATCTGCC-3′
*EGFR*	5′-AATTTACAGGAAATCCTGCATGGC-3′	5′-GATGCTCTCCACGTTGCACA-3′
*Bax*	5′-CATATAACCCCGTCAACGCAG-3′	5′-GCAGCCGCCACAAACATAC-3′
*BAD*	5′-CTTTAAGAAGGGACTTCCTCGCC-3′	5′-AAGTTCCGATCCCACCAGGA-3′
*TGF-β1*	5′-GCCCTGGACACCAACTATTGC-3′	5′-GCTGCACTTGCAGGAGCGCAC-3′
*UCP2*	5′-GGAGGTGGTCGGAGATACCAA-3′	5′-ACAATGGCATTACGAGCAACAT-3′
*18s*	5′-GTAACCCGTTGAACCCCATT-3′	5′-CCATCCAATCGGTAGTAGCG-3′

### Western Blot Analysis

To examine the effects of E_2_ and heteronemin on signaling pathways, Western blot analyses were conducted to quantify protein expression levels of phosphorylated (p)STAT3-S727, p-protein kinase Cα (PKCα)-T497, and pERK1/2 in MCF-7 and MDA-MB-231 cells. For the Western blot analyses, cells were lysed, and extracted protein samples were separated by 10% sodium dodecylsulfate-polyacrylamide gel electrophoresis (SDS-PAGE). A 30-μg quantity of protein was loaded into each well with 5 **×** sample buffer and samples were resolved by electrophoresis at 100 V for 2 h. Resolved proteins were transferred from the polyacrylamide gel to Millipore Immobilon-PSQ Transfer polyvinylidene difluoride membranes (Millipore, Billerica, MA, United States) with Mini Trans-Blot^®^ Cells (Bio-Rad, Hercules, CA, United States). Membranes were blocked with a solution of 2% bovine serum albumin (BSA) in Tris-buffered saline. Membranes were treated with primary antibodies from Cell Signaling Technology: pSTAT3-S727 (catalog no. 9136), pERK1/2 (catalog no. 4377), and GeneTex: pPKCα (catalog no. 130433) and GAPDH (catalog no. 100118). All antibodies were incubated at 4 °C overnight. Proteins were detected with horseradish peroxidase (HRP)-conjugated secondary antibodies and Immobilon^TM^ Western HRP Substrate Luminol Reagent (Millipore, St. Charles, MO, United States). Western blots were visualized and recorded with an Amersham Imager 600 (GE Healthcare Life Sciences, Pittsburgh, PA, United States). Intensities of the protein bands representing expression levels were quantitated using Image J 1.47 software (NIH, Bethesda, MD, United States) according to the software instructions.

### Mitochondrial ROS Assay

Changes in mitochondrial ROS that occurred during apoptosis were detected with a fluorescence-based assay. Mitochondrial ROS was detected with a Mitochondrial Superoxide Detection Kit (ab219943, Abcam, Cambridge, United Kingdom). MCF-7 cells were cultured as previously described. After being starved with 0.25% stripped FBS-containing medium for 2 days, cells were re-fed using 5% stripped FBS-containing medium and treated with E_2_ and heteronemin. Antimycin A at 50 μM was used as a positive control. After 24 h, cells were processed with the Mitochondrial Superoxide Detection Kit according to the manufacturer’s instructions. Excitation at 540 nm and emission at 590 nm was read with a spectral scanning multimode reader (Thermo Fisher Scientific Varioskan Flash, Waltham, MA, United States).

### Cell Cycle Assay

MCF-7 and MDA-MB-231 cells were seeded at a density of 1.5 × 10^5^ cells/well in six-well plates. After starvation with 0.25% stripped FBS-containing medium for 2 days, cells were re-fed using 5% stripped FBS-containing medium and treated with agents for 24 h. Cells were trypsinized and fixed with 70% ethanol and stored at −20°C for 2 weeks prior to propidium iodide (PI) staining and a flow cytometric analysis. Cells were incubated with 1 ml of phosphate-buffered saline (PBS) containing 0.5% Triton X-100 and 0.05% RNase A for 1 h, then stained with PI/RNase Staining Buffer (BD, San Jose, CA, United States) in the dark at room temperature for 30 min. Flow cytometry was carried out on an Invitrogen Attune^TM^ NxT Acoustic Focusing Cytometer (Thermo Fisher Scientific, MA, United States). Percentages of DNA contents were analyzed using Attune NxT Flow Cytometer software (ver. 4.2) to determine the fractions of each phase of the cell cycle (sub G_0_/G_1_, G_0_/G_1_, S, and G_2_/M).

### Statistical Analysis

All collected data for immunoblots, nucleotide densities, and cell densities were analyzed by IBMS^®^PSS^®^ Statistics software vers. 19.0 (SPSS, Chicago, IL, United States). Student’s *t*-test was conducted, and *p*-values of < 0.05 (*,^#^, or ^$^), 0.01 **, ^##^, or ^$$^), and 0.001 (***, ^###^, or ^$$$^) as thresholds of significance, were used to evaluate the significance of effects of estrogen, heteronemin, and their combined treatment.

## Results

### Heteronemin Induces Anti-proliferation and Reverses E_2_ –Induced Proliferation in ER-Positive and ER-Negative Breast Cancer Cells

Heteronemin inhibited cell proliferation in different kinds of cancer cells (Lee Y. S. et al., 2018; Lin et al., 2018; Huang et al., 2020). We examined the inhibitory effect of heteronemin on human ER-positive and ER-negative breast cancer cells. ER-positive MCF-7 cells and ER-negative breast cancer MDA-MB-231 cells were treated with different concentrations of heteronemin that were refreshed daily for 3 days, and a cell viability assay was conducted.

**FIGURE 1:**
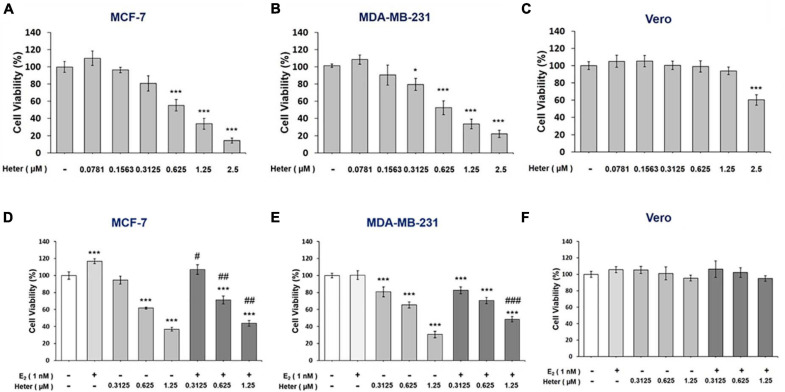
Estrogen reverses heteronemin induces anti-proliferation in breast cancer cells. **(A)** MFC-7, **(B)** MDA-MB-231, and **(C)** Vero cells were treated with various concentrations of heteronemin with medium containing reagents refreshed daily for 72 h. **(D)** MCF-7, **(E)** MDA-MB-231, and **(F)** Vero cells were co-treated with various concentrations of heteronemin in the presence and absence of 1 nM E_2_ for 72 h. Cell viability was detected with an Alamar Blue^®^ Assay Kit. The number of independent studies (*n*) = 4. Results were expressed as the mean ± SD. *p < 0.05, ***p < 0.001 compared to the untreated control. ^#^*p* < 0.05, ^##^*p* < 0.01, ^###^*p* < 0.001, compared to the same dosage of heteronemin treatment only. Heter: Heteronemin.

Heteronemin significantly inhibited the viability of MCF-7 cells at concentrations was higher than 0.625 μM and IC_50_ = 0.8779 μM (Figure 1A). In MDA-MB-231 cells, there was significant inhibition at concentrations higher than 0.3125 μM and IC_50_ = 0.8672 μM (Figure 1B). However, with less toxicity in Vero cells that are the normal kidney epithelial cells extracted from an African green monkey and IC_50_ = 3.5676 μM (Figure 1C). These results suggest that both ER-positive and ER-negative breast cancer cells were sensitive to heteronemin treatment. Moreover, E_2_ induced cell proliferation and reversed anti-proliferation partially induced by heteronemin in MCF-7 cells (Figure 1D). On the other hand, E_2_ did not promote proliferation in MDA-MB-231 cells. However, it slightly reversed heteronemin-induced anti-proliferation at 1.25 μM in MBA-MD-231 cells (Figure 1E). Although E_2_ affect cell proliferation in breast cancer cells, the cell viability were suppressed in the co-treatments. Additionally, combined E2 and heteronemin also do not affect normal cell proliferation (Figure 1E).

### Heteronemin Regulates Gene Expressions Differently in ER-Positive and ER-Negative Breast Cancer Cells

**FIGURE 2:**
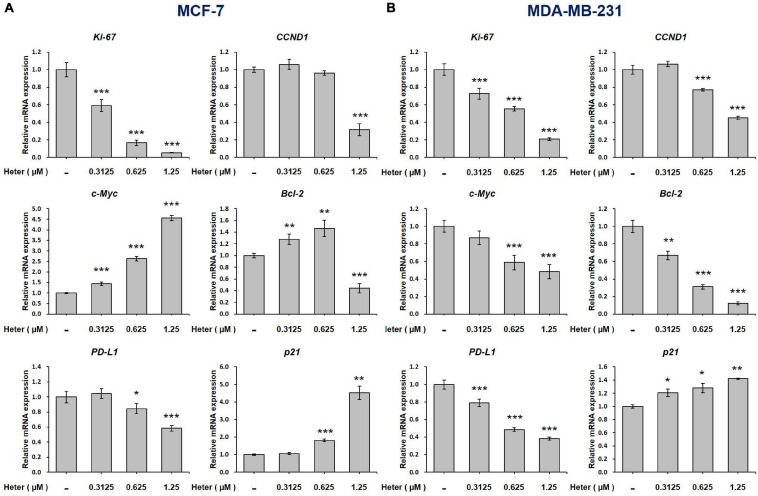
Heteronemin regulates expression of *ki-67*, *CCND1*, *c-Myc, Bcl-2*, *PD-L1*, and *p21* in breast cancer cells. **(A)** MCF-7 and **(B)** MDA-MB-231 cells were treated with various concentrations of heteronemin for 24 h. Total RNA was extracted. qPCR analyses were conducted for *Ki-67, CCND1, c-Myc, Bcl-2*, *PD-L1*, and *p21*. The number of independent studies (*n*) = 3. *p < 0.05, **p < 0.01, ***p < 0.001, compared to the untreated control.

We further studied the effects of heteronemin on gene expressions. Both MCF-7 and MDA-MB-231 cells were treated with different concentrations of heteronemin for 24 h. Cells were harvested and RNA was extracted. qPCR studies were conducted for *Ki-67*, *CCND1*, *c-Myc*, *Bcl-2*, *PD-L1*, and *p21*. Heteronemin significantly inhibited expressions of the proliferation genes, *Ki-67* and *CCND-1*, in concentration-dependent manners in both MCF-7 and MDA-MB-231 cells. It decreased *c-Myc* expression only in MDA-MB-231 cells, while in MCF-7 cells, *c-Myc* expression significantly increased with heteronemin treatment (Figure 2).

Additionally, Heteronemin inhibited programmed death-ligand 1 (PD-L1) expression and stimulated expression of the proapoptotic gene, p21 in dose-dependent manners in both MCF-7 and MDA-MB-231 cells (Figure 2). Heteronemin inhibited *Bcl-2* expression only with a high concentration treatment of 1.25 μM, while *Bcl-2* expression increased at a low concentration in MCF-7 cells. In MDA-MB-231 cells, *Bcl-2* expression was inhibited at all concentrations of heteronemin. It is interesting that heteronemin inhibited expressions of *c-Myc* and *Bcl-2* in ER-negative breast cancer cells but stimulated their expressions in ER-positive breast cancer cells (Figure 2).

### Heteronemin Reverses E_2_ –Induced Gene Expression in Breast Cancer Cells

**FIGURE 3:**
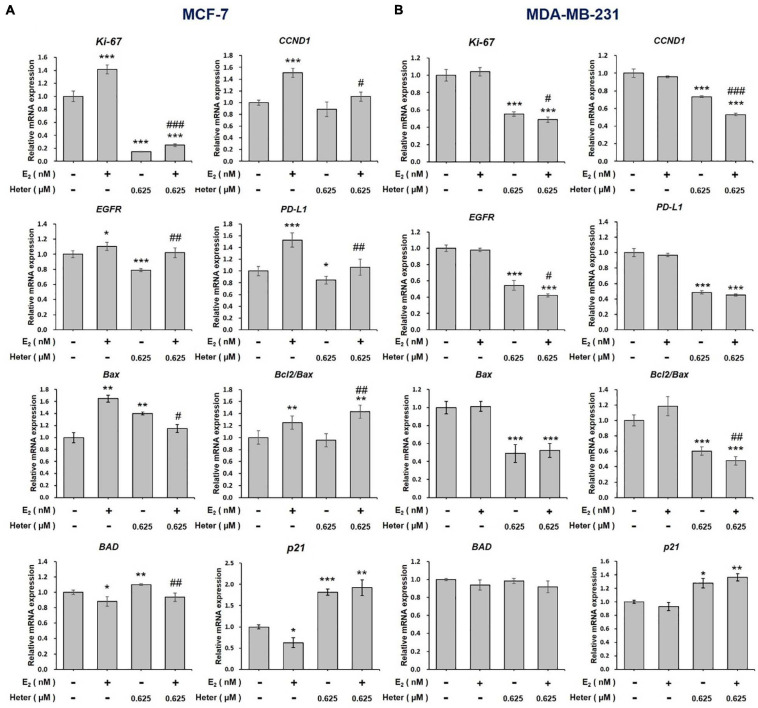
Estrogen and heteronemin regulate expressions of *Ki-67, CCND1, EGFR, PD-L1*, B*ax*, *Bcl/Bax*, *BAD*, and *p21* in breast cancer cells. **(A)** MCF-7 and **(B)** MDA-MB-231 cells were treated with heteronemin (0.625 μM) in the presence and absence of 1 nM E_2_ for 24 h. RNA was extracted and qPCR analyses were conducted for *Ki-67, CCND1, EGFR, PD-L1*, *Bax*, *Bcl-2/Bax*, *BAD*, and *p21*. The number of independent studies (*n*) = 3. *p < 0.05, **p < 0.01, ***p < 0.001, compared to the untreated control. ^#^*p* < 0.05, ^##^*p* < 0.01, ^###^*p* < 0.001, compared to the same dosage of heteronemin treatment only.

We also studied the effect of E_2_ on genes regulated by heteronemin in ER-positive and ER-negative breast cancer cells. In co-treatment, we chose 0.625 and 0.3125 μM heteronemin, respectively, in MCF-7 and MDA-MB-231 cells which exhibited significant inhibition of cell viability (Figure 1). MCF-7 and MDA-MB-231 cells were treated with E_2_, heteronemin, and combined E_2_ and heteronemin for 24 h. E_2_ induced expression of *Ki-67, CCND1, EGFR*, and *PD-L1* significantly in MCF-7 cells. Heteronemin suppressed expression of *Ki-67*, *EGFR*, and *PD-L1* significantly and inhibited E_2_’s stimulatory effect. While 0.625 μM heteronemin treatment did not inhibit *CCND1* expression, it reversed the effect of E_2_ in combined treatment (Figure 3A). In addition, E_2_ not only increased *Bax* expression and the *Bcl-2/Bax* ratio but also inhibited expression of *BAD* and *p21*, two important pro-apoptotic genes in MCF-7 cells (Figure 3A). Heteronemin increased expression of *Bax*, *BAD*, and *p21*. E_2_ reduced the effect and the Bcl-2/Bax ratio increased with combined treatment in MCF-7 cells (Figure 3A).

In ER-negative MDA-MB-231 cells, E_2_ did not affect gene expressions of *Ki-67*, *CCND1, EGFR*, and *PD-L1* (Figure 3B). On the other hand, heteronemin alone or co-treatment with E_2_ suppressed expression of *Ki-67*, *EGFR*, and *PD-L1* (Figure 3B). E_2_ did not affect *Bax*, *BAD*, and *p21* expressions. Heteronemin suppressed expression of *Bax* but stimulated expression of *BAD* and *p21* significantly (Figure 3B). It also reduced *Bcl-2/Bax* ratio. The combined treatment inhibited *Bcl-2/Bax* ratio significantly, but increased expression of *BAD* and *p21* significantly (Figure 3B). In ER-positive MCF-7 cells, E_2_ stimulated expressions of proliferation-related genes and inhibited pro-apoptotic gene expression. Nevertheless, heteronemin could rescue the effects of E_2_. E_2_ did not affect those gene expressions in ER-negative MDA-MB-231 cells. Additionally, results also suggested that *Bcl-2/Bax* ratio may play a vital role in cell fate in MDA-MB-231 cells but not in MCF-7 cells.

### Heteronemin Inhibits Signal Transduction Pathways in Breast Cancer Cells

**FIGURE 4:**
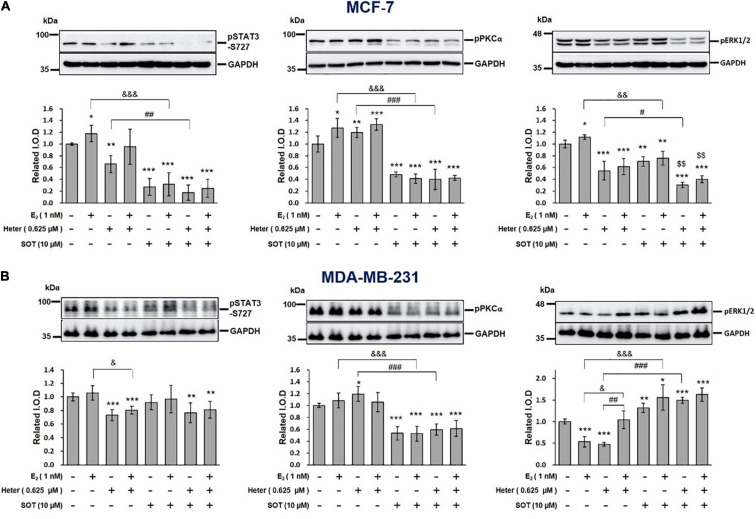
Estrogen, heteronemin, and sotrastaurin (SOT) affect the signal transduction pathway in breast cancer cells. **(A)** MCF-7 and **(B)** MDA-MB-231 cells were treated with 1 nM E_2_, 0.625 μM heteronemin, and their combination in the presence and absence of the PKC inhibitor, SOT for 24 h. Cells were harvested and total proteins were extracted. Western blot analyses were conducted for phosphorylated (p)STAT3, pPKCα, and pERK1/2. The number of independent studies (*n*) = 4. *p < 0.05, **p < 0.01, ***p < 0.001, compared to the untreated control. ^#^*p* < 0.05, ^##^*p* < 0.01, ^###^*p* < 0.001 compared to heteronemin. ^&^*p* < 0.05, ^&⁣&^*p* < 0.01, ^&⁣&⁣&^*p* < 0.001, compared to E_2_.

We investigated the mechanisms involved in the heteronemin-induced anti-cancer ability in breast cancer cells. Previous studies showed that activation of STAT3, ERK1/2, and PKC plays an important role in proliferation in cancer cells (Lønne et al., 2009; Nana et al., 2018; Chin et al., 2019; Huang et al., 2020). Results shown E_2_ induced phosphorylation of ERK1/2, PKCα, and STAT3. Alternatively, heteronemin inhibited activation of ERK1/2 and STAT3, but it increased PKCα phosphorylation in MCF-7 cancer cells (Figure 4). The increased phosphorylated STAT3 and ERK1/2 induced by E_2_ was reversed by heteronemin. In the presence of a PKC inhibitor, sotrastaurin (SOT), all activities of ERK1/2, PKCα, and STAT3 were inhibited in MCF-7 cells. Heteronemin enhanced the inhibitory effect of SOT on activities of ERK1/2 and STAT3 (Figure 4).

In MDA-MB-231 cells, E_2_ did not affect STAT3 and PKCα phosphorylation. Heteronemin treatment at 0.625 μM suppressed the phosphorylation of STAT3 and ERK1/2 but slightly increased PKCα activation. Interestingly, E_2_ reduced the inhibition of pERK1/2 but did not reduce the inhibition of pSTAT3 induced by heteronemin (Figure 4). The result of PKCα was similar from MCF-7 cells, in which SOT just suppressed PKCα activation.

### Both E_2_ and Heteronemin Induce Mitochondrial ROS Production in MCF-7 Cells

**FIGURE 5:**
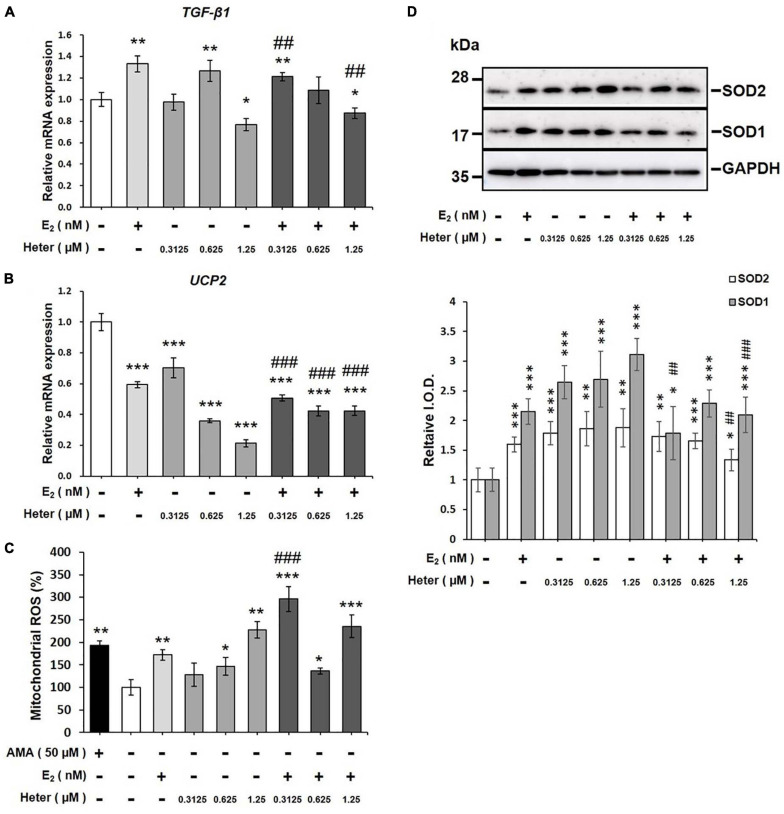
Estrogen and heteronemin regulate expressions of *TGF-β1* and *UCP2* and induce mitochondrial reactive oxygen species (ROS) production in MCF-7 cancer cells. Cells were treated with different concentrations of heteronemin in the presence and absence of 1 nM E_2_ for 24 h. Cells were harvested and analyzed for **(A)** TGF-β1 expression, **(B)** *UCP2* expression, **(C)** mitochondrial ROS production, and **(D)** SODs accumulation. The number of independent studies (*n*) = 4. *p < 0.05, **p < 0.01, ***p < 0.001, compared to the untreated control. ^##^*p* < 0.01, ^###^*p* < 0.001, compared to the same dosage of heteronemin treatment only.

Activation of signal transduction is linked to ROS production. To investigate whether E_2_ or heteronemin plays a direct role in mitochondrial function, we performed a mitochondrial ROS assay. MCF-7 cells were treated with E_2_ and different concentrations of heteronemin for 24 h. E_2_ increased *TGF-β1* expression which has been shown to increase ROS production (Joo et al., 2008) but downregulated *UCP2* expression (Figures 5A, B). On the other hand, heteronemin stimulated *TGF-β1* expression at 0.625 μM but inhibited its expression at 1.25 μM (Figure 5A). Expression of *UCP2* was inhibited by heteronemin in a concentration-dependent manner (Figure 5B). The stimulatory effect of E_2_ on *TGF-β1* was inhibited by heteronemin treatment (Figure 5A). The inhibitory effect of E_2_ on *UCP2* was further enhanced by heteronemin treatment (Figure 5B). Not only did E_2_ significantly increase mitochondrial ROS production, but heteronemin also significantly increased mitochondrial ROS production in a dose-dependent manner (Figure 5C). In the presence of E_2_, 0.3125 μM heteronemin increased mitochondrial ROS production compared to heteronemin only (Figure 5C). Interestingly, both E_2_ and heteronemin increased accumulation of superoxide dismutase (SOD)s (Figure 5D). However, the combination of E_2_ and heteronemin reversed the accumulation of SOD1 and, SOD2 was reversed only at the higher concentration heteronemin (Figure 5D).

### Heteronemin Induces Sub G_0_/G_1_ Increase and Arrests Cell Cycle at G_0_/G_1_ Phase to Block Cell Proliferation

**FIGURE 6:**
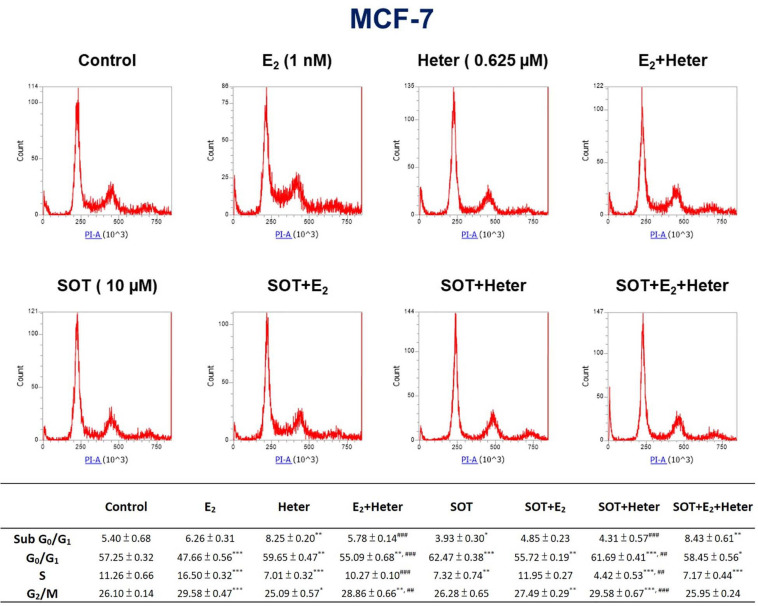
Estrogen, heteronemin, and sotrastaurin (SOT) affect proliferation that induces apoptosis and cell cycle arrest in breast cancer cells. MCF-7 cells were treated with 1 nM E_2,_ 0.625 μM heteronemin in the presence and absence of 10 μM SOT for 24 h. Cells were harvested, and a flow cytometric assay was conducted as described in section “Materials and Methods.” *n* = 3. *p < 0.05, **p < 0.01, ***p < 0.001, compared to the untreated control. ^##^*p* < 0.01, ^###^*p* < 0.001, compared to heteronemin treatment only.

To understand whether E_2_ or heteronemin is involved in regulating cell death in breast cancer cells, we performed propidium iodide (PI) staining for further exploration. Percentages of the various cell phases in MCF-7 cells are shown in Figure 6. Cells were treated with E_2_ and 0.625 μM heteronemin in the presence and absence of SOT for 24 h. The results indicated that G_0_/G_1_ phase was reduced and S phase and G_2_/M phase were increased with E_2_ treatment. Heteronemin treatment not only increased sub G_0_/G_1_ and G_0_/G_1_ phase population but also decreased S phase. Compared with heteronemin treatment, co-treatment of E_2_ and heteronemin decreased sub G_0_/G_1_ and G_0_/G_1_ phase but increased S phase. These data suggest that heteronemin caused cell apoptosis, G_0_/G_1_ arrest, and then reduced cell proliferation. In addition, heteronemin could reverse the effects induced by E_2_. SOT, an inhibitor of PKC, enhanced the G_0_/G_1_ phase increasing and S phase decreasing by heteronemin.

In summary, heteronemin inhibited activation of ERK1/2 and STAT3 via different pathways in MCF-7 cells and MDA-MB-231 cells. The former was PKC-dependent. Heteronemin also inhibited proliferation-related gene expressions, increased proapoptotic gene expressions, and suppressed cell viability. Bcl-2/Bax ratio was downgraded in MDA-MB-231 cells but no change in MCF-7 cells after heteronemin treatment. Heteronemin was able to increase mitochondrial ROS production and SODs accumulation in MCF-7 cells. In the presence of E_2_, ROS production was increased but SODs were decreased to reverse heteronemin-induced anti-proliferative effects. Thus, E_2_ rescued heteronemin-induced anti-proliferative effects in both MCF-7 and MDA-MB-231 breast cancer cells via different mechanisms.

## Discussion

Heteronemin caused anti-proliferation via cell apoptosis and cell cycle arrest in breast cancer cell lines are observed (Figures 1, 6). Heteronemin inhibited expressions of *Ki-67*, *CCND-1*, and *PD-L1* in concentration-dependent manners and stimulated *p21* expression in both MCF-7 and MDA-MB-231 cells (Figure 2). These results confirmed our recent observations in oral cancer cells (Huang et al., 2020) and another report (Kopf et al., 2013). On the other hand, heteronemin inhibited expressions of *c-Myc* and *Bcl-2* in MDA-MB-231 cells but stimulated their expression in MCF-7 cells (Figure 2). In addition, E_2_ stimulated expression of those genes and reversed some inhibitory effects of heteronemin (Figure 3). Heteronemin reduced *Bcl-2/Bax* ratio in MDA-MB-231 cells but not in MCF-7 cells (Figure 3). Heteronemin also affected the protein expressions of the Bcl-2 family (Supplementary Figure 1), In MCF-7 cells, heteronemin increased *Bcl-2, Bax*, and *Bid* expression. However, Bcl-2 expression was suppressed by heteronemin in MDA-MB-231. Thus, *Bcl-2/Bax* ratio was downgraded in MDA-MB-231 cells but no change in MCF-7 cells after heteronemin treatment. E_2_ inhibited stimulatory effects of heteronemin and the *Bcl-2/Bax* ratio increased with combined treatment (Figure 3A). In MDA-MB-231 cells, E_2_ did not stimulate *Bax* expression although suppressed *BAD* expression slightly (Figure 3B). These results suggested that the ratio of *Bcl-2/Bax* should play a critical role in determining fate in MDA-MB-231 cells but not in MCF-7 cells. On the other hand, E_2_ activated ERK1/2, PKCα, and STAT3, and reversed effects of heteronemin on activation of *Bcl-2* and reduction of BAD. Therefore, increased *Bcl-2* expression in MCF-7 cells but reduced *Bcl-2* expression in MDA-MB-231 cells by heteronemin may reflect different mechanisms in different types of breast cancer cells.

E_2_ treatment can stimulate PKC activation through ERα followed by the activation of PKCδ or via ERα directly upregulating PKC (Ronda et al., 2010). Therefore, activated PKC plays an important role in estrogen-stimulated breast cancer cell proliferation. In addition, the PKC inhibitor, AEB071 (sotrastaurin), was shown to be effective against triple-negative breast cancer cells (Byerly et al., 2016). Heteronemin repressed STAT3 (Wu et al., 2016). Heteronemin inhibited activation of STAT3 and ERK1/2 (Figure 4). Sotrastaurin inhibits Akt phosphorylation, NF-κB/STAT3 activation, and Mcl-1 upregulation. It also renders cells sensitive to arsenic trioxide (Amigo-Jiménez et al., 2015). Therefore, we used sotrastaurin to block PKC to reduce the NF-κB/STAT3 signaling pathway and investigated mechanisms in breast cancer cells.

Estrogen reversed the inhibitory effect of heteronemin against activation of ERK1/2 and STAT3 (Figure 4) in MCF-7 cancer cells and activation of PKC and STAT3 in MDA-MB-231 cancer cells (Figure 4) but diminished ERK1/2 in MDA-MB-231 cancer cells. These results suggested that E_2_ activates different signaling pathways in MCF-7 and MDA-MB-231 cancer cells. Activation of E_2_-induced PKC may mediate E_2_-dependent biological activities including cell proliferation. The PKC inhibitor, sotrastaurin, more completely inhibited STAT3 activation than did heteronemin in MCF-7 cells (Figure 4), however, but not in MDA-MB-231 cells (Figure 4). Estradiol binds to the αvβ3 integrin (Cody et al., 2007), and this possibly explains the result that cell viability of MDA-MB-231 triple-negative breast cancer cells was affected by E_2_.

Heteronemin is a farnesyl transferase inhibitor (FTI) that inhibits the cytarabine-induced, farnesyl transferase-mediated Ras activation and inhibits Ras downstream signal transduction pathways such as mitogen-activated protein kinases (MAPKs), activator protein (AP)-1, nuclear factor (NF)-κB, and c-Myc (Saikia et al., 2018). Overproduced ROS by conjugated estrogens during estrogen metabolism activates IκB kinase (IKK)-α and -β to increase the translocation of nuclear NF-κB (Kim et al., 2000). Alterations of mitochondrial metabolism may induce ROS overproduction to involve in estrogen-mediated carcinogenesis via induction of oxidative DNA damage (Tian et al., 2016). Additionally, blocking estrogen attenuates respiratory and metabolic responses and superoxide accumulation in estrogen-responsive breast cancer cells (Tan et al., 2002; Doan et al., 2004). Oxidative stress was postulated to be one of the mechanisms underlying E_2_’s carcinogenic effect in breast cancer. E_2_ increases mitochondrial-derived ROS by an unknown mechanism (Sastre-Serra et al., 2010). E_2_ significantly increased ROS production in MCF-7 cells (Figure 5C). UCPs regulate energy efficiency in mitochondria and production of ROS (Chouchani et al., 2016; Pierelli et al., 2017; Cadenas, 2018). They function as an adaptive anti-oxidant defense to protect against over-productive oxidation (Pierelli et al., 2017). E_2_ downregulated *UCP* expression and significantly increased mitochondrial ROS production in ER-positive MCF-7 cells (Figure 5). Heteronemin inhibited *UCP2* expression in a dose-dependent manner (Figure 5B). Heteronemin also significantly increased ROS production at 0.625 and 1.25 μM (Figure 5C). Lu’s group showed that heteronemin treatment (2.56 μM) increased ROS levels in LNCaP cells (Lee Y. S. et al., 2018). These results suggest that heteronemin may suppress UCP2 accumulation to increase ROS production. UCP2 was shown to negatively modulate intracellular ROS production (Pierelli et al., 2017). In the presence of E_2_, 0.3125 μM heteronemin increased ROS production compared to heteronemin only (Figure 5C). Combined treatment with E_2_ and heteronemin consistently suppressed *UCP2* expression (Figure 5B), suggesting that downregulation of UCP2 may play an important role in heteronemin-induced anti-proliferation in ER-positive breast cancer cells in the presence of estradiol. Overall, these results suggest that through an ER-dependent mechanism, E_2_ may increase mitochondrial ROS production by repressing UCPs, which offers a new perspective on the understanding of why E_2_ is a risk factor for breast cancer. Heteronemin stimulated *TGF-β1* expression at 0.625 μM but inhibited its expression at 1.25 μM (Figure 5A). The stimulatory effect of E_2_ on *TGF*-β was inhibited by heteronemin treatment (Figure 5A). The inhibitory effect of E_2_ on *UCP2* was further enhanced by heteronemin treatment (Figure 5B). Downregulation of UCP2 expression by TGF-β-SMAD4 signaling was shown to play a regulatory role in mitochondrial ROS formation (Kim and Lee, 2018). Thus, heteronemin stimulated TGF-β1 expression to downregulate UCP expression and further increase ROS production in MCF-7 cells at 0.625 μM.

It is not surprising to observe that E_2_ increased SODs accumulation (Figure 5D) as reported (Rao et al., 2008). On the other hand, anti-oxidant also stimulates ROS production in previous studies (Heo et al., 2018; Kim et al., 2019). Both ceramide and resveratrol stimulate mitochondrial potential. Thus, at certain concentrations, heteronemin may increase SOD to activate mitochondrial potential (Figure 5). However, the combination of heteronemin and E_2_ decreased SOD accumulation to reach the balance between oxidation and anti-oxidation.

The ROS scavenger, N-acetyl cysteine (NAC), inhibited heteronemin-induced mitochondrial ROS production and cell apoptosis (Lin et al., 2018). Heteronemin significantly increased both cellular ROS and mtROS. It also induced the loss of the mitochondrial membrane potential (MMP) in lung cancer cells (Cheng et al., 2019). It increased the percentage of apoptotic cells and ROS in Molt4 cells (Chen et al., 2018). Heteronemin -treated lung cancer cells showed a significant increase in both cellular ROS and mtROS, which in turn caused the loss of the MMP. Heteronemin decreased expressions of the anti-oxidant enzymes Cu/ZnSOD, MnSOD, and catalase (Cheng et al., 2019). Pretreatment with the mitochondrion-targeted anti-oxidant, MitoTEMPO, reduced heteronemin-induced apoptosis through a mitochondrion-dependent apoptotic pathway, which was accompanied by increased cell viability, decreased mtROS, enhanced MMP, and suppressed expressions of cleaved caspase-3 and caspase-9 proteins (Cheng et al., 2019). Oxidative phosphorylation performed in mitochondria and glycolysis in the cytoplasm were inhibited, which subsequently reduced downstream ATP production (Cheng et al., 2019). Additionally, Chang et al., 2021 reported heteronemin may with high toxicity and causing animal death (Chang et al., 2021). However, Lee M. G. et al. (2018) also shown heteronemin can using safely with a lower dosage.

These results suggest heteronemin inhibited activation of ERK1/2 and STAT3 in both ER-positive and ER-negative breast cancer cells. In addition, heteronemin downregulated the expression of *Ki-67*, *CCND1*, *EGFR*, and *PD-L1*, but upregulated *p21* and *BAD* expression. However, *Bcl-2/Bax* ratio was downgraded in MDA-MB-231 cells but no change in MCF-7 cells after heteronemin treatment. It restrained *UCP2* expression, extended ROS production, increased SODs accumulation, induced G_0_/G_1_ arrest, and caused anti-proliferation in breast cancer MCF-7 cells. On the other hand, E_2_ activated ERK1/2, PKC, and STAT3, increased ROS production, and recued heteronemin-induced biological activities.

## Conclusion

**FIGURE 7:**
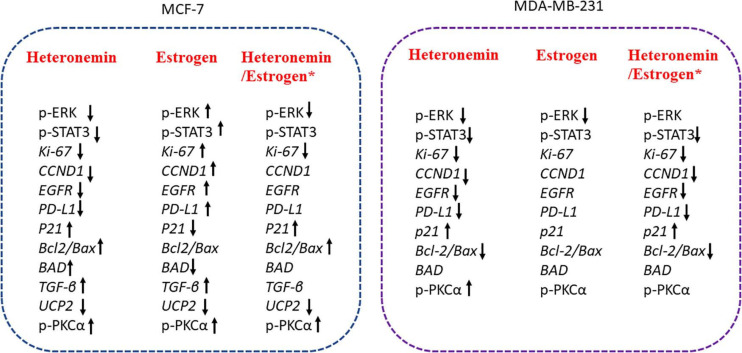
The schematic of heteronemin reverses E_2_–induced mechanism in breast cancer cells. *Significantly different compare with the untreated control group. Heteronemin: 0.625 μM, E_2_: 1nM.

In conclusion, heteronemin inhibited ER-positive and ER-negative breast cancer cell proliferation via different mechanisms (Figure 7), but less effect on normal cells. Additionally, heteronemin also could overcome E2 stimulated proliferation. Those results suggest Heteronemin had a strong capacity to inhibit proliferation in both MCF-7 and MDA-MB-231 breast cancer cells. Thus, heteronemin has a potential as an anti-cancer drug.

## Data Availability Statement

The original contributions presented in the study are included in the article/Supplementary Material, further inquiries can be directed to the corresponding author/s.

## Author Contributions

Y-CY, M-CL, and H-YL contributed the study concept and methodology design of this study. Z-LL, T-YH, and H-YC conducted the experiments. Z-LL, K-WS, and H-YC analyzed and interpreted the data. Y-CY and H-YL wrote the manuscript. Z-LL, H-YC, T-YH, C-YL, H-MH, S-YL, JW-P, PD, and KW helped in revising the manuscript. All authors contributed to the article and approved the submitted version.

## Conflict of Interest

The authors declare that the research was conducted in the absence of any commercial or financial relationships that could be construed as a potential conflict of interest.

## Publisher’s Note

All claims expressed in this article are solely those of the authors and do not necessarily represent those of their affiliated organizations, or those of the publisher, the editors and the reviewers. Any product that may be evaluated in this article, or claim that may be made by its manufacturer, is not guaranteed or endorsed by the publisher.

## References

[B1] Amigo-Jiménez I. Bailón E. Aguilera-Montilla N. Terol M. J. García-Marco J. A. García-Pardo A. (2015). Bone marrow stroma-induced resistance of chronic lymphocytic leukemia cells to arsenic trioxide involves Mcl-1 upregulation and is overcome by inhibiting the PI3Kδ or PKCβ signaling pathways. *Oncotarget* 6 44832–44848. 26540567 10.18632/oncotarget.6265 PMC4792595

[B2] Anampa J. Makower D. Sparano J. A. (2015). Progress in adjuvant chemotherapy for breast cancer: an overview. *BMC Med.* 13:195. 10.1186/s12916-015-0439-8 26278220 PMC4538915

[B3] Bhat A. H. Dar K. B. Anees S. Zargar M. A. Masood A. Sofi M. A. (2015). Oxidative stress, mitochondrial dysfunction and neurodegenerative diseases; a mechanistic insight. *Biomed. Pharmacother.* 74 101–110. 26349970 10.1016/j.biopha.2015.07.025

[B4] Bray F. Ferlay J. Soerjomataram I. Siegel R. L. Torre L. A. Jemal A. (2018). Global cancer statistics 2018: GLOBOCAN estimates of incidence and mortality worldwide for 36 cancers in 185 countries. *CA Cancer J. Clin.* 68 394–424. 10.3322/caac.21492 30207593

[B5] Byerly J. Halstead-Nussloch G. Ito K. Katsyv I. Irie H. Y. (2016). PRKCQ promotes oncogenic growth and anoikis resistance of a subset of triple-negative breast cancer cells. *Breast Cancer Res.* 18:95. 10.1186/s13058-016-0749-6 PMC5034539 27663795

[B6] Cadenas S. (2018). Mitochondrial uncoupling, ROS generation and cardioprotection. *Biochim. Biophys. Acta Bioenerg.* 1859 940–950. 10.1016/j.bbabio.2018.05.019 29859845

[B7] Cavalieri E. Rogan E. (2014). The molecular etiology and prevention of estrogen-initiated cancers: Ockham’s Razor: pluralitas non est ponenda sine necessitate. Plurality should not be posited without necessity. *Mol. Aspects Med.* 36 1–55. 10.1016/j.mam.2013.08.002 23994691 PMC3938998

[B8] Cavalieri E. L. Stack D. E. Devanesan P. D. Todorovic R. Dwivedy I. Higginbotham S. (1997). Molecular origin of cancer: catechol estrogen-3,4-quinones as endogenous tumor initiators. *Proc. Natl. Acad. Sci. U.S.A.* 94 10937–10942. 10.1073/pnas.94.20.10937 9380738 PMC23537

[B9] Chang W. T. Bow Y. D. Fu P. J. Li C. Y. Wu C. Y. Chang Y. H. (2021). A marine terpenoid, heteronemin, induces both the apoptosis and ferroptosis of hepatocellular carcinoma cells and involves the ROS and MAPK pathways. *Oxid. Med. Cell. Longev.* 2021:7689045. 10.1155/2021/7689045 PMC7803406 33488943

[B10] Chen Y. C. Lu M. C. El-Shazly M. Lai K. H. Wu T. Y. Hsu Y. M. (2018). Breaking down leukemia walls: heteronemin, a sesterterpene derivative, induces apoptosis in leukemia Molt4 cells through Oxidative stress, mitochondrial dysfunction and induction of talin expression. *Mar, Drugs* 16:212. 10.3390/md16060212 29914195 PMC6025351

[B11] Cheng M. H. Huang H. L. Lin Y. Y. Tsui K. H. Chen P. C. Cheng S. Y. (2019). BA6 induces apoptosis via stimulation of reactive oxygen species and inhibition of oxidative phosphorylation in human lung cancer cells. *Oxid. Med. Cell. Longev.* 2019:6342104. 10.1155/2019/6342104 PMC6530211 31205586

[B12] Chin Y. T. He Z. R. Chen C. L. Chu H. C. Ho Y. Su P. Y. (2019). Tetrac and NDAT induce anti-proliferation via integrin αvβ3 in colorectal cancers with different K-RAS status. *Front. Endocrinol.* 10:130. 10.3389/fendo.2019.00130 PMC6422911 30915033

[B13] Chipuk J. E. Kuwana T. Bouchier-Hayes L. Droin N. M. Newmeyer D. D. Schuler M. (2004). Direct activation of Bax by p53 mediates mitochondrial membrane permeabilization and apoptosis. *Science* 303 1010–1014. 10.1126/science.1092734 14963330

[B14] Chouchani E. T. Kazak L. Jedrychowski M. P. Lu G. Z. Erickson B. K. Szpyt J. (2016). Mitochondrial ROS regulate thermogenic energy expenditure and sulfenylation of UCP1. *Nature* 532 112–116. 10.1038/nature17399 27027295 PMC5549630

[B15] Cobbaut M. Van Lint J. (2018). Function and regulation of protein kinase d in oxidative stress: a tale of isoforms. *Oxid. Med. Cell. Longev.* 2018:2138502. 10.1155/2018/2138502 PMC5944262 29854077

[B16] Cody V. Davis P. J. Davis F. B. (2007). Molecular modeling of the thyroid hormone interactions with alpha v beta 3 integrin. *Steroids* 72 165–170. 10.1016/j.steroids.2006.11.008 17166537

[B17] Devanesan P. Santen R. J. Bocchinfuso W. P. Korach K. S. Rogan E. G. Cavalieri E. (2001). Catechol estrogen metabolites and conjugates in mammary tumors and hyperplastic tissue from estrogen receptor-alpha knock-out (ERKO)/Wnt-1 mice: implications for initiation of mammary tumors. *Carcinogenesis* 22 1573–1576. 10.1093/carcin/22.9.1573 11532882

[B18] Doan V. D. Gagnon S. Joseph V. (2004). Prenatal blockade of estradiol synthesis impairs respiratory and metabolic responses to hypoxia in newborn and adult rats. *Am. J. Physiol. Regul. Integr. Comp. Physiol.* 287 R612–R618. 15142837 10.1152/ajpregu.00627.2003

[B19] el-Deiry W. S. Harper J. W. O’connor P. M. Velculescu V. E. Canman C. E. Jackman J. (1994). WAF1/CIP1 is induced in p53-mediated G1 arrest and apoptosis. *Cancer Res.* 54 1169–1174. 8118801

[B20] Fussell K. C. Udasin R. G. Smith P. J. Gallo M. A. Laskin J. D. (2011). Catechol metabolites of endogenous estrogens induce redox cycling and generate reactive oxygen species in breast epithelial cells. *Carcinogenesis* 32 1285–1293. 10.1093/carcin/bgr109 21665890 PMC3149209

[B21] Gwangwa M. V. Joubert A. M. Visagie M. H. (2019). Effects of glutamine deprivation on oxidative stress and cell survival in breast cell lines. *Biol. Res.* 52:15. 10.1186/s40659-019-0224-9 PMC6437944 30917872

[B22] Heo J. R. Kim S. M. Hwang K. A. Kang J. H. Choi K. C. (2018). Resveratrol induced reactive oxygen species and endoplasmic reticulum stress-mediated apoptosis, and cell cycle arrest in the A375SM malignant melanoma cell line. *Int. J. Mol. Med.* 42 1427–1435. 29916532 10.3892/ijmm.2018.3732 PMC6089775

[B23] Hofseth L. J. Raafat A. M. Osuch J. R. Pathak D. R. Slomski C. A. Haslam S. Z. (1999). Hormone replacement therapy with estrogen or estrogen plus medroxyprogesterone acetate is associated with increased epithelial proliferation in the normal postmenopausal breast. *J. Clin. Endocrinol. Metab.* 84 4559–4565. 10.1210/jc.84.12.4559 10599719

[B24] Huang C. H. Huang T. Y. Chang W. J. Pan Y. S. Chu H. R. Li Z. L. (2020). Combined treatment of heteronemin and tetrac induces antiproliferation in oral cancer cells. *Mar. Drugs* 18:348. 10.3390/md18070348 32630719 PMC7401260

[B25] Joo C. K. Kim H. S. Park J. Y. Seomun Y. Son M. J. Kim J. T. (2008). Ligand release-independent transactivation of epidermal growth factor receptor by transforming growth factor-beta involves multiple signaling pathways. *Oncogene* 27 614–628. 10.1038/sj.onc.1210649 17637750

[B26] Kim D. W. Sovak M. A. Zanieski G. Nonet G. Romieu-Mourez R. Lau A. W. (2000). Activation of NF-kappaB/Rel occurs early during neoplastic transformation of mammary cells. *Carcinogenesis* 21 871–879. 10.1093/carcin/21.5.871 10783306

[B27] Kim T. H. Park J. H. Woo J. S. (2019). Resveratrol induces cell death through ROS-dependent downregulation of Notch1/PTEN/Akt signaling in ovarian cancer cells. *Mol. Med. Rep.* 19 3353–3360. 30816513 10.3892/mmr.2019.9962

[B28] Kim Y. H. Lee S. H. (2018). TGF-β/SMAD4 mediated UCP2 downregulation contributes to *Aspergillus* protease-induced inflammation in primary bronchial epithelial cells. *Redox Biol.* 18 104–113. 10.1016/j.redox.2018.06.011 30007886 PMC6067066

[B29] Kollmann Z. Bersinger N. A. Mckinnon B. D. Schneider S. Mueller M. D. Von Wolff M. (2015). Anti-Müllerian hormone and progesterone levels produced by granulosa cells are higher when derived from natural cycle IVF than from conventional gonadotropin-stimulated IVF. *Reprod. Biol. Endocrinol.* 13:21. 10.1186/s12958-015-0017-0 PMC4379743 25889012

[B30] Kopf S. Viola K. Atanasov A. G. Jarukamjorn K. Rarova L. Kretschy N. (2013). In vitro characterisation of the anti-intravasative properties of the marine product heteronemin. *Arch. Toxicol.* 87 1851–1861. 10.1007/s00204-013-1045-1 23543012

[B31] Lee M. G. Liu Y. C. Lee Y. L. El-Shazly M. Lai K. H. Shih S. P. (2018). Heteronemin, a marine sesterterpenoid-type metabolite, induces apoptosis in prostate LNcap cells via oxidative and ER stress combined with the inhibition of topoisomerase II and Hsp90. *Mar. Drugs* 16:204. 10.3390/md16060204 29890785 PMC6025191

[B32] Lee Y. S. Chin Y. T. Shih Y. J. Nana A. W. Chen Y. R. Wu H. C. (2018). Thyroid hormone promotes beta-catenin activation and cell proliferation in colorectal cancer. *Horm. Cancer* 9 156–165. 10.1007/s12672-018-0324-y 29380230 PMC10355916

[B33] Lin H. Y. Tey S. L. Ho Y. Chin Y. T. Wang K. Whang-Peng J. (2018). Heteronemin induces anti-proliferation in cholangiocarcinoma cells via inhibiting TGF-β pathway. *Mar. Drugs* 16:489. 10.3390/md16120489 30563284 PMC6316595

[B34] Lønne G. K. Masoumi K. C. Lennartsson J. Larsson C. (2009). Protein kinase Cdelta supports survival of MDA-MB-231 breast cancer cells by suppressing the ERK1/2 pathway. *J. Biol. Chem.* 284 33456–33465. 10.1074/jbc.m109.036186 19833733 PMC2785190

[B35] Nana A. W. Wu S. Y. Yang Y. S. Chin Y. T. Cheng T. M. Ho Y. (2018). Nano-diamino-tetrac (NDAT) enhances resveratrol-induced antiproliferation by action on the RRM2 pathway in colorectal cancers. *Horm. Cancer* 9 349–360. 10.1007/s12672-018-0334-9 30027502 PMC10355899

[B36] Okoh V. O. Felty Q. Parkash J. Poppiti R. Roy D. (2013). Reactive oxygen species via redox signaling to PI3K/AKT pathway contribute to the malignant growth of 4-hydroxy estradiol-transformed mammary epithelial cells. *PLoS One* 8:e54206. 10.1371/journal.pone.0054206 23437041 PMC3578838

[B37] Papi A. Orlandi M. Bartolini G. Barillari J. Iori R. Paolini M. (2008). Cytotoxic and antioxidant activity of 4-methylthio-3-butenyl isothiocyanate from *Raphanus sativus* L. (Kaiware Daikon) sprouts. *J Agric Food Chem* 56 875–883. 10.1021/jf073123c 18189352

[B38] Pierelli G. Stanzione R. Forte M. Migliarino S. Perelli M. Volpe M. (2017). Uncoupling protein 2: a key player and a potential therapeutic target in vascular diseases. *Oxid. Med. Cell. Longev.* 2017:7348372. 10.1155/2017/7348372 PMC5661070 29163755

[B39] Poillet-Perez L. Despouy G. Delage-Mourroux R. Boyer-Guittaut M. (2015). Interplay between ROS and autophagy in cancer cells, from tumor initiation to cancer therapy. *Redox Biol.* 4 184–192. 10.1016/j.redox.2014.12.003 25590798 PMC4803791

[B40] Rao A. K. Ziegler Y. S. Mcleod I. X. Yates J. R. Nardulli A. M. (2008). Effects of Cu/Zn superoxide dismutase on estrogen responsiveness and oxidative stress in human breast cancer cells. *Mol. Endocrinol.* 22 11 13–1124. 10.1210/me.2007-0381 PMC2366184 18258688

[B41] Ronda A. C. Buitrago C. Boland R. (2010). Role of estrogen receptors, PKC and Src in ERK2 and p38 MAPK signaling triggered by 17β-estradiol in skeletal muscle cells. *J. Steroid Biochem. Mol. Biol.* 122 287–294. 10.1016/j.jsbmb.2010.05.002 20478382

[B42] Saikia M. Retnakumari A. P. Anwar S. Anto N. P. Mittal R. Shah S. (2018). Heteronemin, a marine natural product, sensitizes acute myeloid leukemia cells towards cytarabine chemotherapy by regulating farnesylation of Ras. *Oncotarget* 9 18115–18127. 10.18632/oncotarget.24771 29719594 PMC5915061

[B43] Sastre-Serra J. Valle A. Company M. M. Garau I. Oliver J. Roca P. (2010). Estrogen down-regulates uncoupling proteins and increases oxidative stress in breast cancer. *Free Radic. Biol. Med.* 48 506–512. 10.1016/j.freeradbiomed.2009.11.025 19969066

[B44] Schumacher M. Cerella C. Eifes S. Chateauvieux S. Morceau F. Jaspars M. (2010). Heteronemin, a spongean sesterterpene, inhibits TNF alpha-induced NF-kappa B activation through proteasome inhibition and induces apoptotic cell death. *Biochem. Pharmacol.* 79 610–622. 10.1016/j.bcp.2009.09.027 19814997

[B45] Tan D. J. Bai R. K. Wong L. J. (2002). Comprehensive scanning of somatic mitochondrial DNA mutations in breast cancer. *Cancer Res.* 62 972–976. 11861366

[B46] Tian H. Gao Z. Wang G. Li H. Zheng J. (2016). Estrogen potentiates reactive oxygen species (ROS) tolerance to initiate carcinogenesis and promote cancer malignant transformation. *Tumour Biol.* 37 141–150. 10.1007/s13277-015-4370-6 26566628

[B47] Weng C. J. Yang Y. T. Ho C. T. Yen G. C. (2009). Mechanisms of apoptotic effects induced by resveratrol, dibenzoylmethane, and their analogues on human lung carcinoma cells. *J. Agric. Food Chem.* 57 5235–5243. 10.1021/jf900531m 19441815

[B48] Wu J. C. Wang C. T. Hung H. C. Wu W. J. Wu D. C. Chang M. C. (2016). Heteronemin is a novel c-Met/STAT3 inhibitor against advanced prostate cancer cells. *Prostate* 76 1469–1483. 10.1002/pros.23230 27416770

[B49] Wu S. Y. Sung P. J. Chang Y. L. Pan S. L. Teng C. M. (2015). Heteronemin, a spongean sesterterpene, induces cell apoptosis and autophagy in human renal carcinoma cells. *Biomed. Res. Int.* 2015:738241. 10.1155/2015/738241 PMC4450260 26090440

[B50] Xu Y. Ge R. Du J. Xin H. Yi T. Sheng J. (2009). Corosolic acid induces apoptosis through mitochondrial pathway and caspase activation in human cervix adenocarcinoma HeLa cells. *Cancer Lett.* 284 229–237. 10.1016/j.canlet.2009.04.028 19457606

