# Supplementary figures and images for: Effect of Estrogen on Heteronemin-Induced Anti-proliferative Effect in Breast Cancer Cells With Different Estrogen Receptor Status
Source: Front Cell Dev Biol. 2021 Jul 26;9:688607. doi: 10.3389/fcell.2021.688607 (PMC8350732; doi:10.3389/fcell.2021.688607)

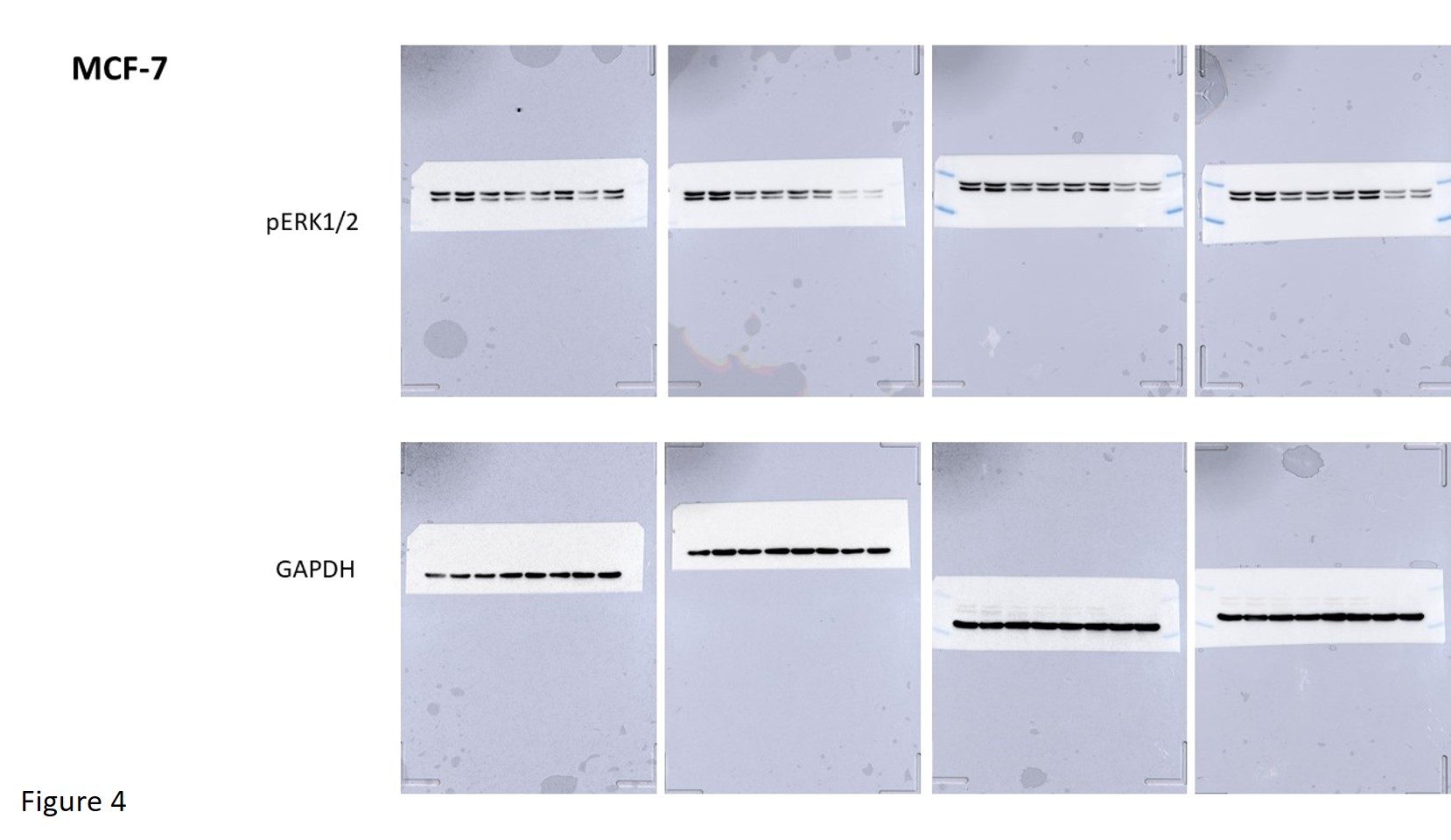

Supplement: Supplementary file 1 [file Data_Sheet_1.ZIP › original picture of WB-0622/Fig. 4-MCF-7-pERK.JPG]

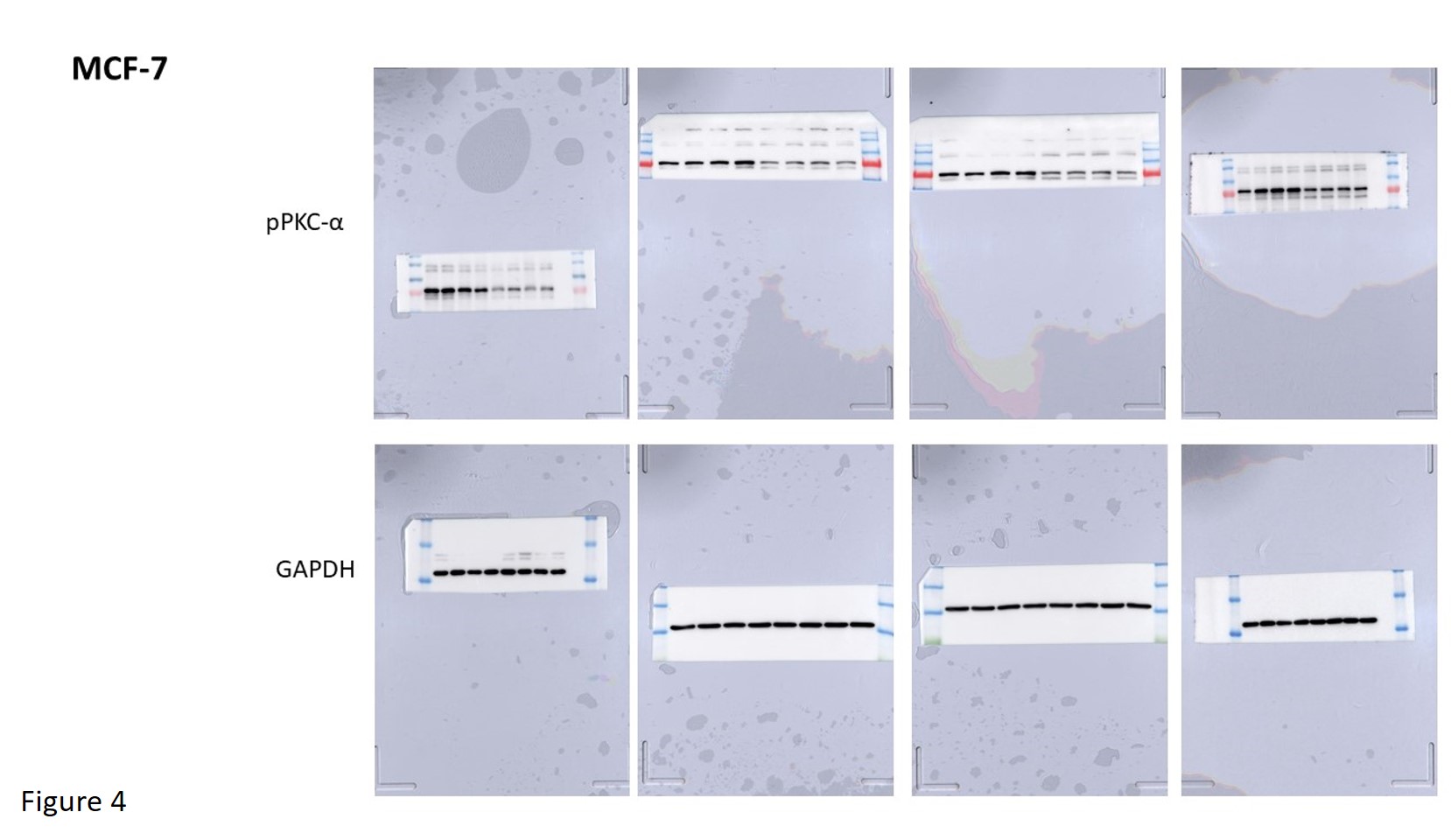

Supplement: Supplementary file 1 [file Data_Sheet_1.ZIP › original picture of WB-0622/Fig. 4-MCF-7-pPKC.JPG]

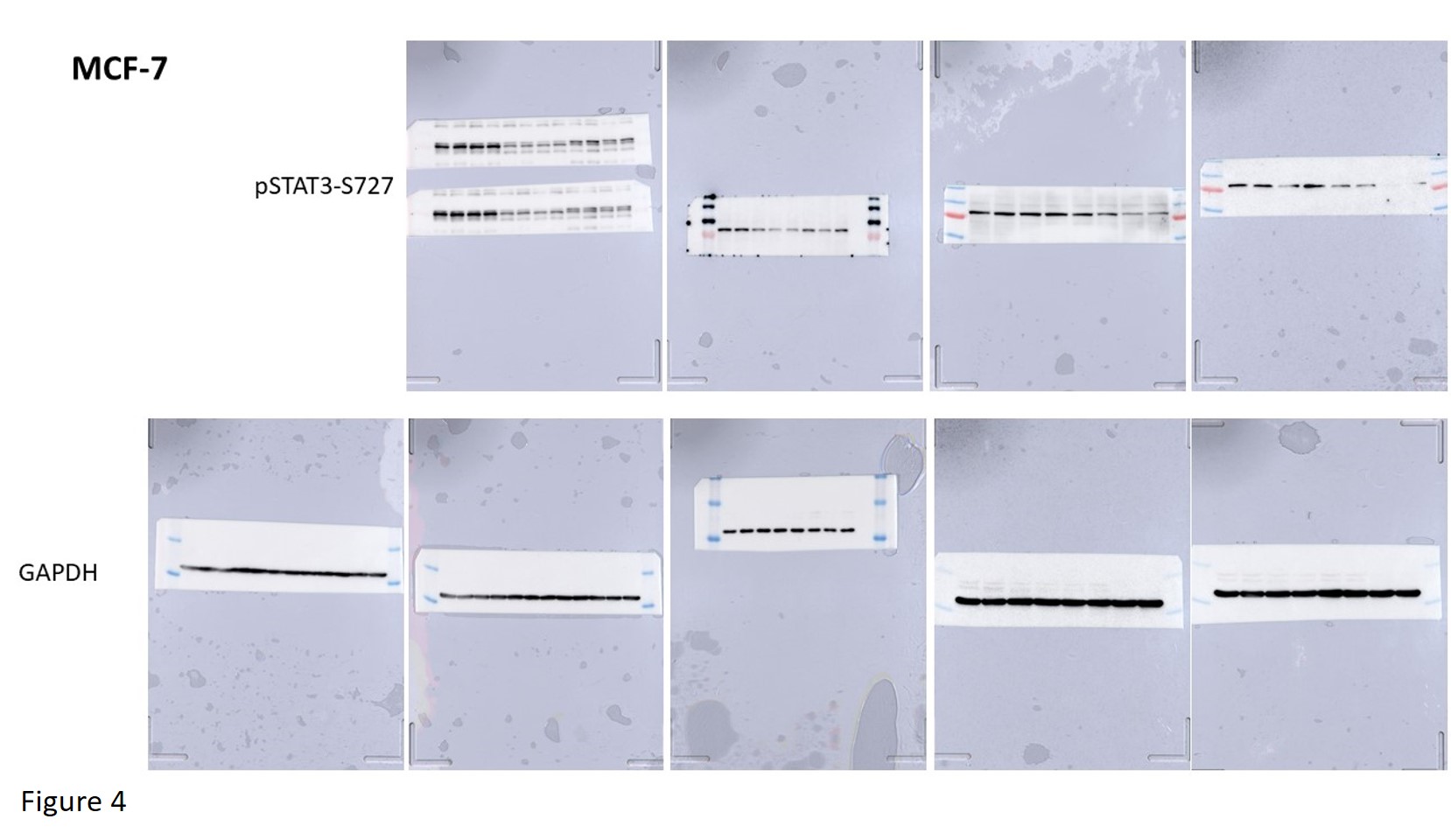

Supplement: Supplementary file 1 [file Data_Sheet_1.ZIP › original picture of WB-0622/Fig. 4-MCF-7-pSTAT3.JPG]

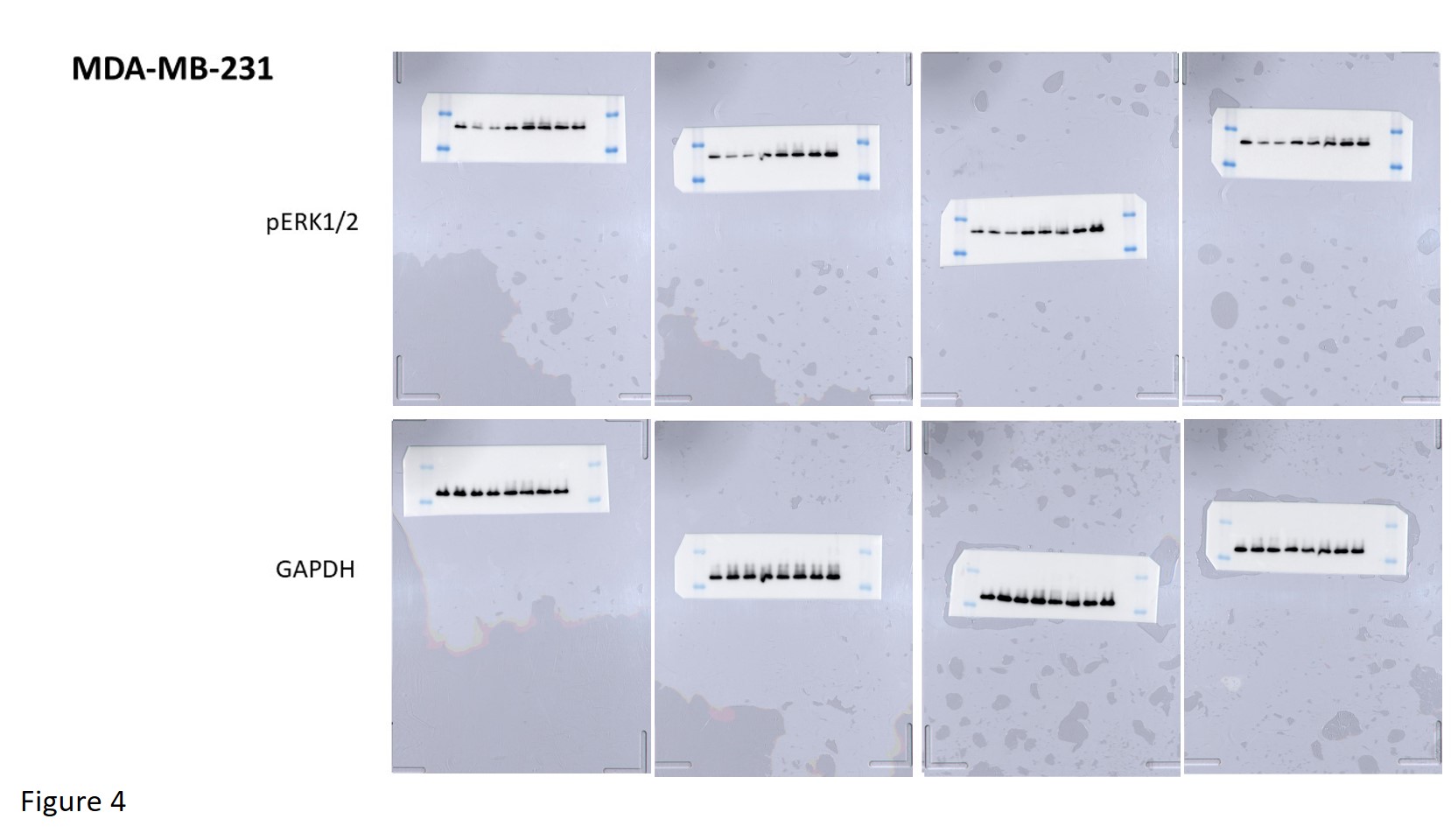

Supplement: Supplementary file 1 [file Data_Sheet_1.ZIP › original picture of WB-0622/Fig. 4-MDA-MB-231-pERK.JPG]

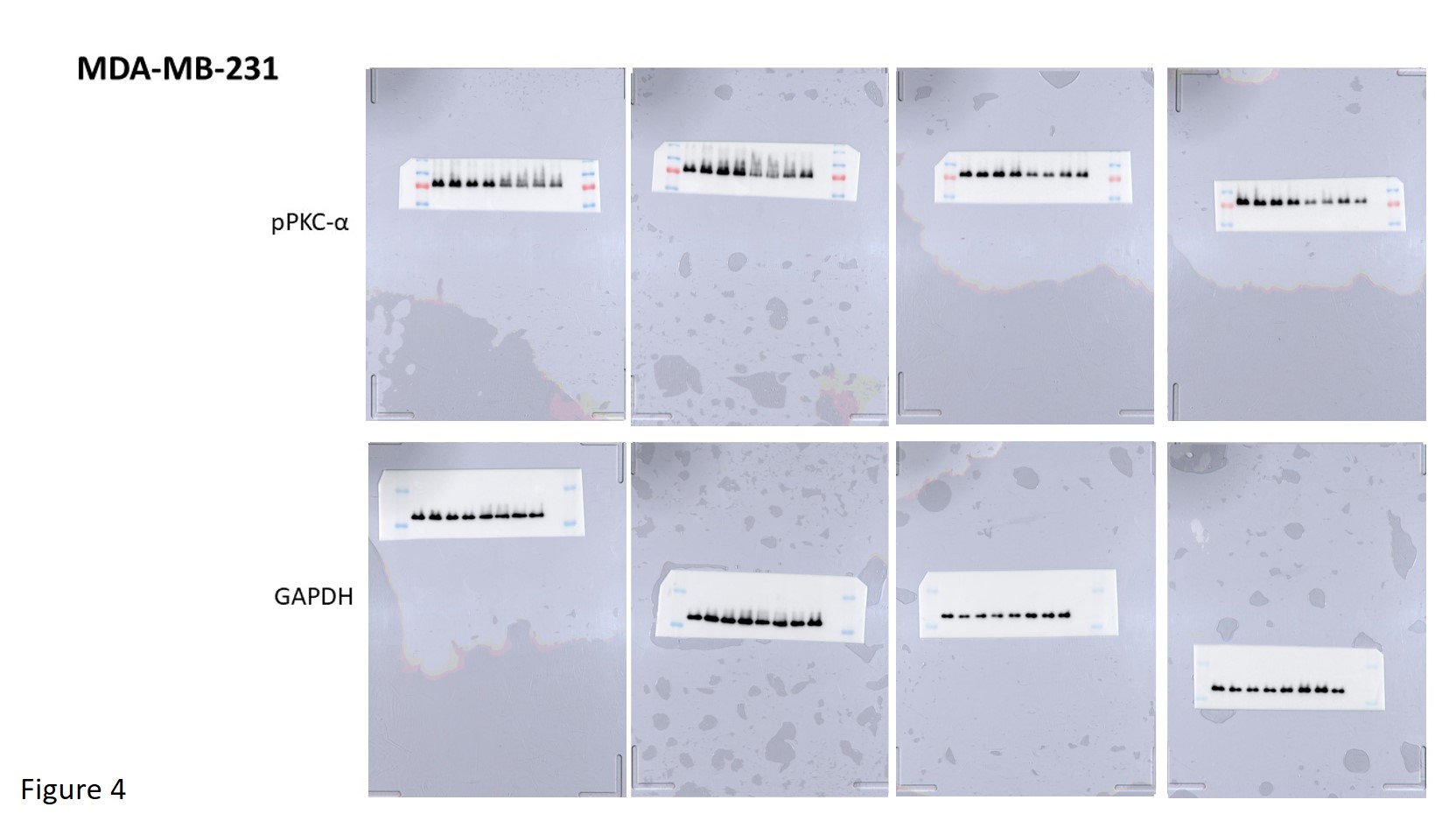

Supplement: Supplementary file 1 [file Data_Sheet_1.ZIP › original picture of WB-0622/Fig. 4-MDA-MB-231-pPKC.JPG]

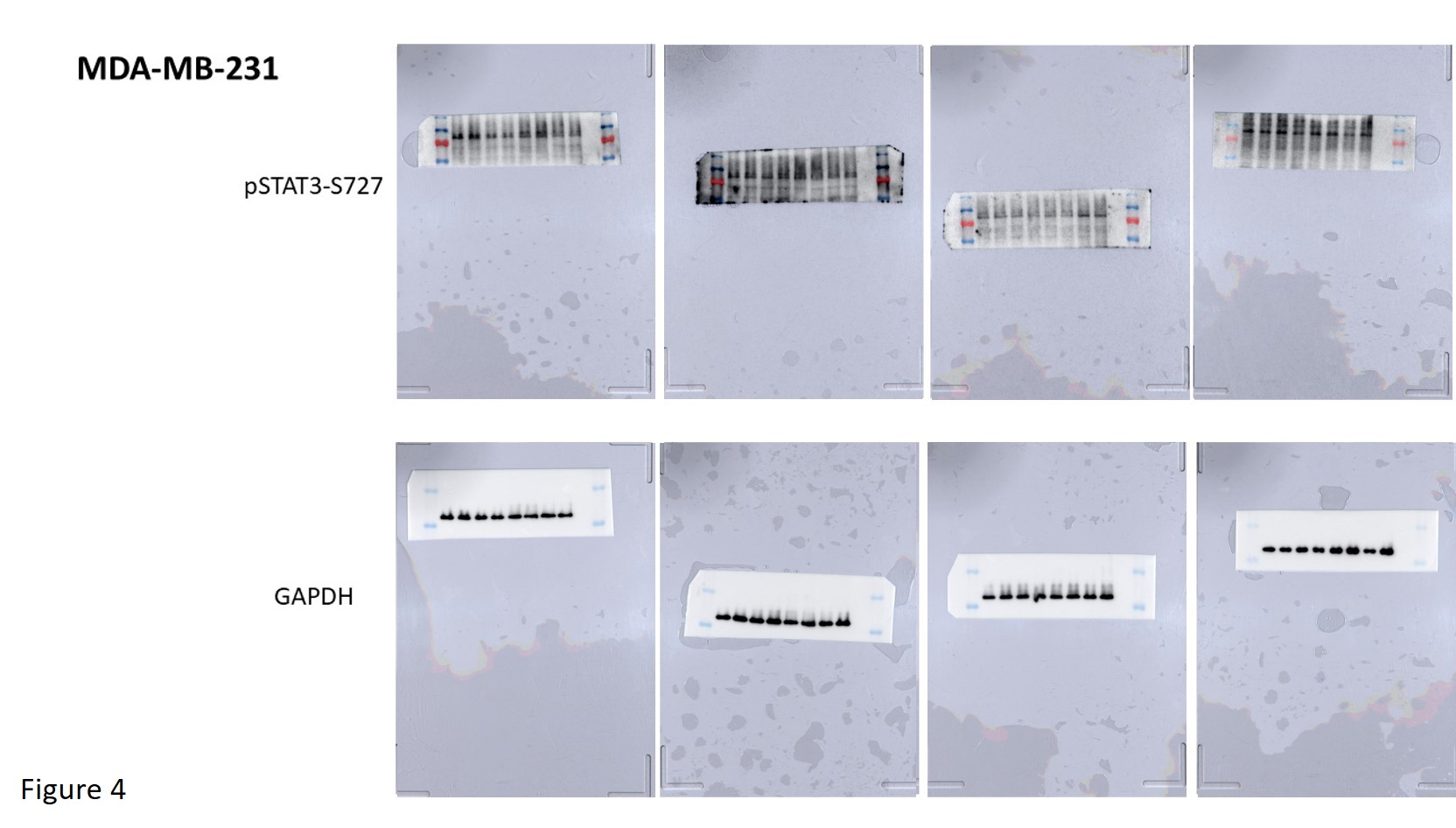

Supplement: Supplementary file 1 [file Data_Sheet_1.ZIP › original picture of WB-0622/Fig. 4-MDA-MB-231-pSTAT3.JPG]

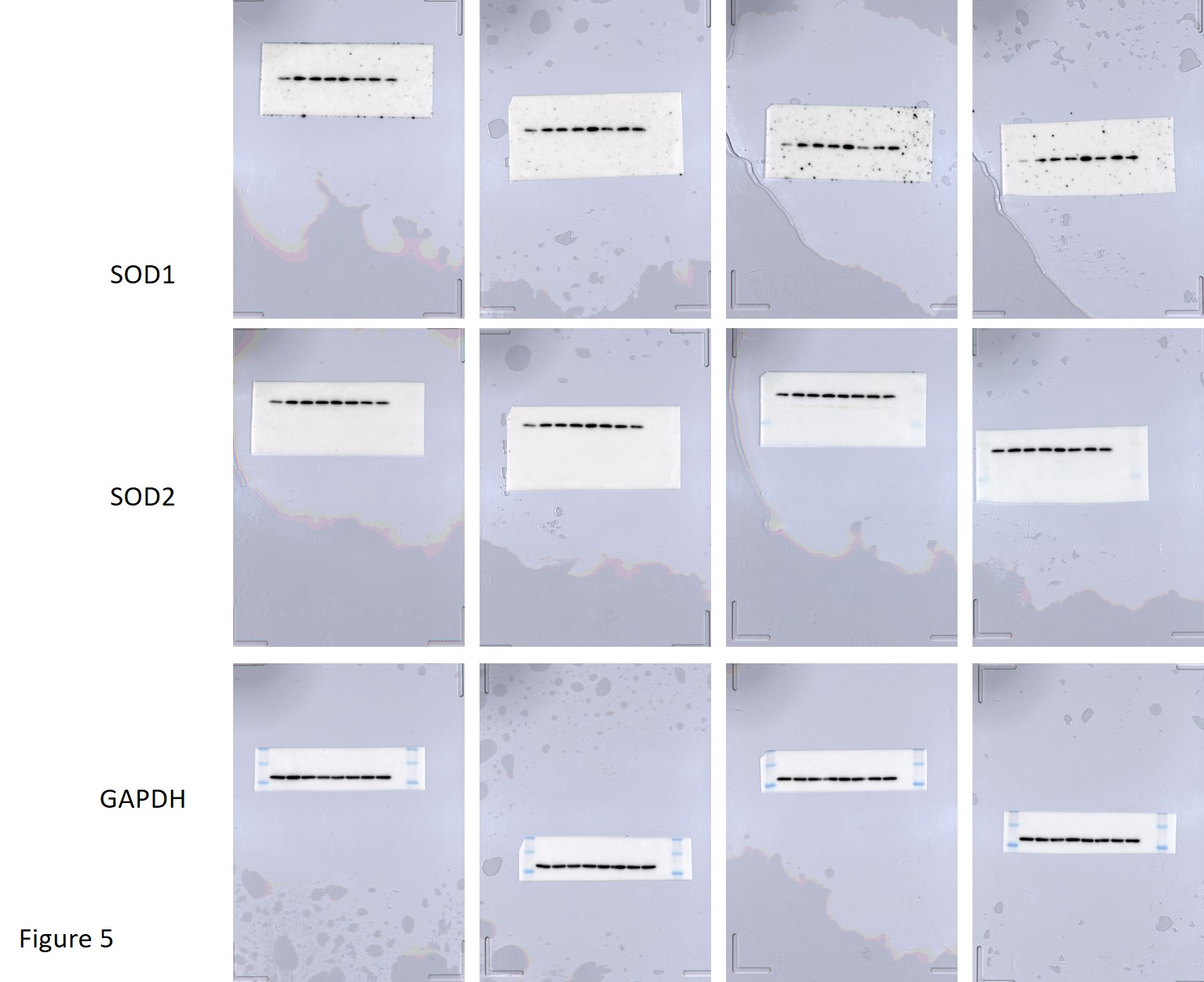

Supplement: Supplementary file 1 [file Data_Sheet_1.ZIP › original picture of WB-0622/Fig. 5-SODs.jpg]

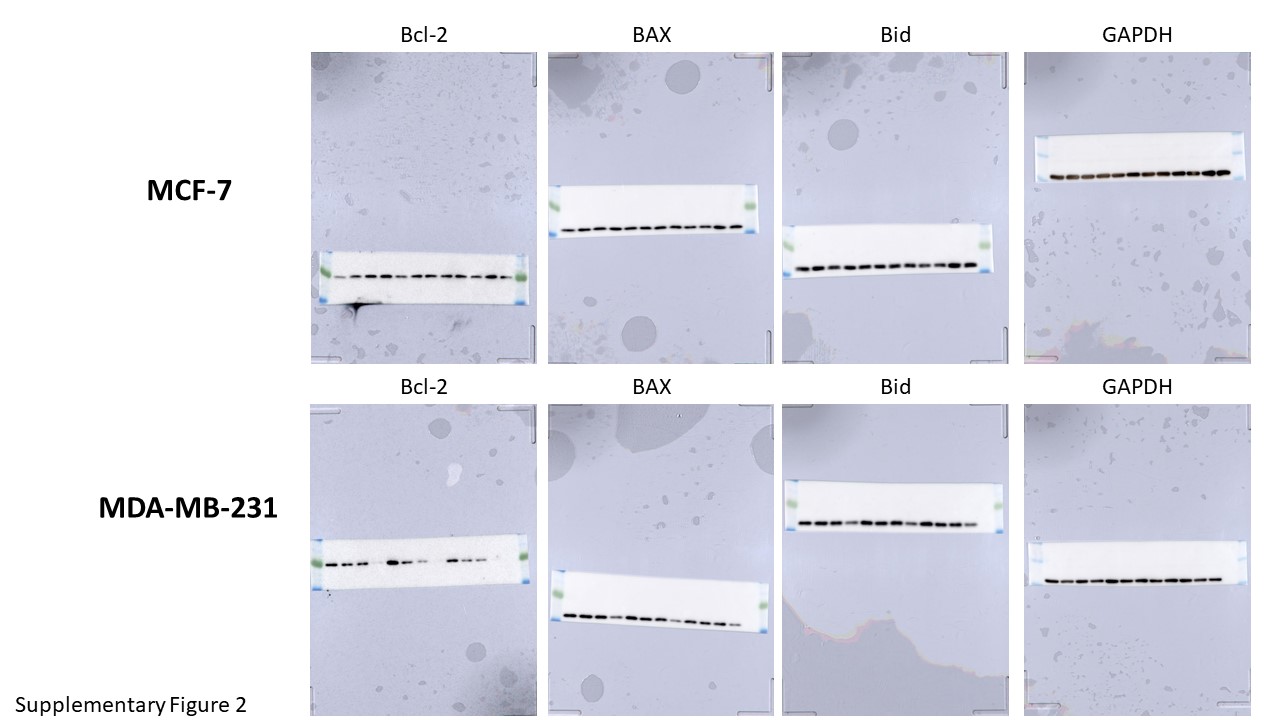

Supplement: Supplementary file 1 [file Data_Sheet_1.ZIP › original picture of WB-0622/Supplementary Figure 1.JPG]
